# Phosphorylation profile of human AQP2 in urinary exosomes by LC–MS/MS phosphoproteomic analysis

**DOI:** 10.1007/s10157-020-01899-4

**Published:** 2020-06-11

**Authors:** Masaki Sakai, Keiko Yamamoto, Hiroaki Mizumura, Tomoki Matsumoto, Yasuko Tanaka, Yumi Noda, Kenichi Ishibashi, Tadashi Yamamoto, Sei Sasaki

**Affiliations:** 1 Department of Pathophysiology, Meiji Pharmaceutical University, 2-522-1 Noshio, Kiyose, Tokyo, 204-8588 Japan; 2 Biofluid Biomarker Center, Niigata University, 8050 Ikarashi 2-no-cho, Nishi-ku, Niigata, 950-2181 Japan; 3 Department of Nephrology, Nitobe Memorial Nakano General Hospital, Tokyo, 164-8607 Japan; 4 Department of Nephrology, Tokyo Medical and Dental University, Tokyo, 113-8519 Japan

**Keywords:** Aquaporin-2, Urine concentration, Proteomics, Kidney, Spectrometry

## Abstract

**Background:**

Aquaporin-2 (AQP2) is a key water channel protein which determines the water permeability of the collecting duct. Multiple phosphorylation sites are present at the C-terminal of AQP2 including S256 (serine at 256 residue), S261, S264 and S/T269, which are regulated by vasopressin (VP) to modulate AQP2 trafficking. As the dynamics of these phosphorylations have been studied mostly in rodents, little is known about the phosphorylation of human AQP2 which has unique T269 in the place of S269 of rodent AQP2. Because AQP2 is excreted in urinary exosomes, the phosphoprotein profile of human AQP2 can be easily examined through urinary exosomes without any intervention.

**Methods:**

Human urinary exosomes digested with trypsin or glutamyl endopeptidase (Glu-C) were examined by the liquid chromatography coupled with tandem mass spectrometry (LC–MS/MS) phosphoproteomic analysis.

**Results:**

The most dominant phosphorylated AQP2 peptide identified was S256 phosphorylated form (pS256), followed by pS261 with less pS264 and far less pT269, which was confirmed by the western blot analyses using phosphorylated AQP2-specific antibodies. In a patient lacking circulating VP, administration of a VP analogue showed a transient increase (peak at 30–60 min) in excretion of exosomes with pS261 AQP2.

**Conclusion:**

These data suggest that all phosphorylation sites of human AQP2 including T269 are phosphorylated and phosphorylations at S256 and S261 may play a dominant role in the urinary exosomal excretion of AQP2.

## Introduction

AQP2 water channel is a key membrane protein which determines the water permeability of kidney collecting duct allowing adjustment of urine concentrating ability [1, 2]. Multiple phosphorylation sites exist in the C-terminal of AQP2 including S256, S261, S264 and S/T269 [3, 4]. Interestingly, the residue at 269 is S in rodents but T in humans [5]. In animal models and culture cells, the phosphorylation at S269 is stimulated by vasopressin (VP), which has been shown to be an important signal for apical membrane accumulation of AQP2 [6, 7]. However, it is unclear whether T269 in human AQP2 is similarly phosphorylated as is the case with S269 in rodent AQP2. Another phosphorylation site, S256, is strongly phosphorylated and remains mostly unchanged even after VP stimulation, while the amount of intracellular pS261-AQP2 decreases after VP treatment [3, 8, 9]. The decrease of pS261-AQP2 will be caused by dephosphorylation, degradation, or even extrusion out of the cell, which remains to be clarified.

AQP2 is excreted into the urine through the endocytosis-multiple vesicular body (MVB)-exosome pathway [10–12], whose physiological significance, however, is not clear. Moreover, it is also unclear whether phosphorylation is necessary for exosomal excretion of AQP2. In this study, human urinary exosomes were applied to the LC–MS/MS (liquid chromatography coupled to tandem mass spectrometry) phosphoproteomic analysis to examine the profile of AQP2 phosphorylation. Moreover, urinary exosomes obtained from a patient with central diabetes insipidus (CDI) were analyzed for the status of AQP2 phosphorylation before and after VP treatment.

## Material and methods

### Urine exosome preparation

Exosomes were prepared from urine samples by an ultracentrifugation method [12]. In brief, urine samples were centrifuged at 3,000 × *g* for 15 min at 25 °C to remove sediments, debris and cells. Then, the supernatants were further centrifuged at 17,000 × *g* for 15 min at 25 °C to remove larger vesicles. The supernatants were finally ultracentrifuged at 160,000 or 200,000 × *g* for 1 h at 25 °C to collect exosomes. The exosomes were suspended in phosphate buffered saline for further analysis. In human, urine samples were collected from the first urine in the morning from healthy volunteers who had given written informed consent (Ethical Committee of Meiji Pharmaceutical University; approved number 2609). Mouse urine samples were obtained from the stored urine collected by metabolic cages.

### Phosphoproteomic analysis of AQP2 in urine exosomes

Three exosome samples from different individuals were digested with trypsin (Agilent, USA) or Glu-C (Promega) in solution and purified by C18 column (GL Sciences, Tokyo, Japan) as reported previously [12], and then each sample was analyzed in triplicate by LC–MS/MS (Bruker nanoElute UHPLC—Bruker timsTOF pro, 115 min gradient method). The proteins were identified by Mascot search engine (v2.3.1, Matrix Science) with search parameters; variable modifications: phosphorylation (ST), peptide mass tolerance: 50 ppm, fragment mass tolerance: 0.05 Da, max missed cleavages: 2, false discovery rate: < 1%, protein database: Uniprot-Swissprot (*n* = 20,386).

### Immunoblotting

Urine exosome samples were denatured in SDS sample-buffer (Nacalai Tesque, Kyoto, Japan) for 20 min at 75 °C. Then, the samples were separated by SDS-PAGE and the proteins were transferred to a PVDF membrane (Immobilon-P, Merk KGaA, Darmstadt, Germany). The blots were probed with following primary antibodies: rabbit anti AQP2 antibody for total AQP2 [5], rabbit anti pS256-AQP2 antibody (Abcam, Cambridge, UK) [13, 14], rabbit anti pS261-AQP2 antibody (PhosphoSolutions, Aurora, USA) [14], rabbit anti pS264-AQP2 antibody (PhosphoSolutions) [15] and rabbit anti pS269-AQP2 antibody (PhosphoSolutions) [14]. Alkaline-phosphatase-conjugated anti rabbit IgG antibody (Promega, Madison, USA) was used as a secondary antibody and Western blue (Promega) was used to detect the signals [13]. The band intensities of the western blots were quantified using ImageJ software (https://imagej.nih.gov/ij/).

### The protocol for a central diabetes insipidus patient

Urine samples were obtained from a central diabetes insipidus patient (CDI, 45 years old, female). As a part of the regular treatment, VP analogue, dDAVP (deamino-Cys1, D-Arg8-vasopressin) 0.25 µg was administered to the nasal mucosa after a 24 h withdrawal period. Urine samples were collected pre and after the administration until 3 h. The patient had given written informed consent (Ethical Committee of Nitobe Memorial Nakano General Hospital; approved number 16-045).

The serum creatinine concentration and osmolality of urine samples were measured by an autoanalyzer and a freezing-point osmometer (Micro-Osmometer, Model 210, Fiske, USA), respectively. The amount of AQP2 protein in urine samples were measured by a sandwich enzyme linked immunosorbent assay (ELISA) method (Otsuka Pharmaceutical Co., Japan) as previously reported [16]. The samples were pre-treated with alkali (0.3 N NaOH for 20 min) before the assay to disrupt exosome membranes [16].

## Results

### Phosphoproteomic analysis of AQP2 in urine exosomes of healthy volunteers

To examine the phosphorylation profile of AQP2 in urinary exosomes, LC–MS/MS was employed in triplicate for three trypsin-digested normal human urinary exosome samples. In each analysis 0.2 µg peptide was applied. One trial showed a total of 112,924 PSMs (protein sequence matches) for 2926 proteins were identified, of which AQP2-match PSMs were 122. Among these 122 PMSs, the phosphorylated were 67, indicating that about half of AQP2 peptides were phosphorylated at any S (167, 182, 256, 261, 264) or T (179, 244, 269) more detailed in Table 1. In the C-terminal of AQP2, almost all PMSs were terminated at R267 by trypsin and the phosphorylation at S256 was frequently observed. Of note, a single PMS (Q_255_SVELHSPQSLPRGTKA_271_) probably generated by the miss-cleavage of trypsin had triple phosphorylation sites at S256, S264 and T269, which indicates that T269 in human AQP2 is phosphorylated as is the case with S269 in rodent AQP2. The results of three studies are summated and shown in Table 2. The total number of the phosphorylation was 185, which was composed of 83% pS256, 8% pS261 and 2% pS264. The phosphorylation at T269 was observed only twice (1%).

**Table 1:**
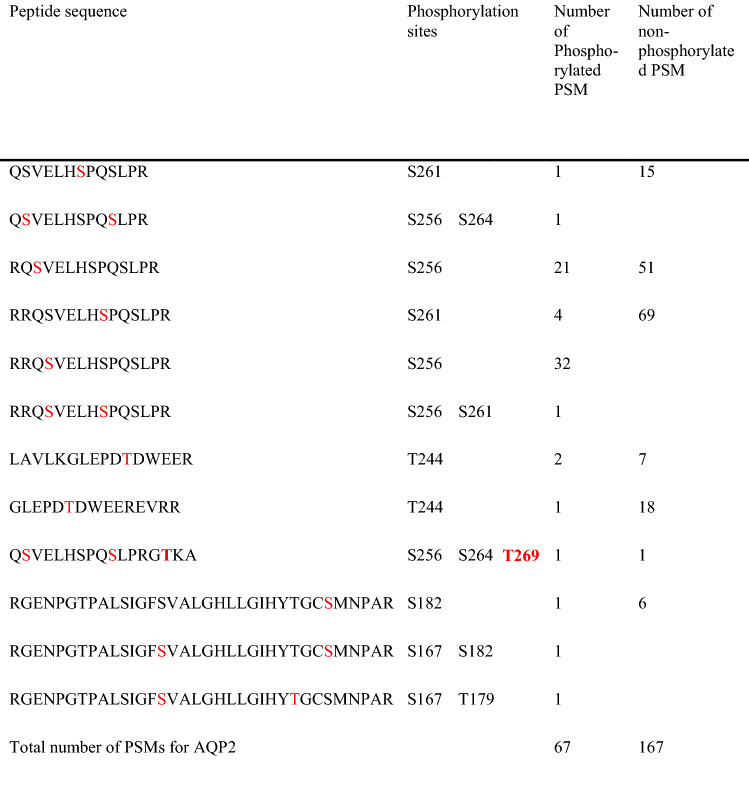
The numbers of phosphorylated and non-phosphorylated AQP2 peptides

Human urine exosomes were digested by trypsin and applied to LC–MS/MS. Numbers of phosphorylated and non-phosphorylated AQP2 match PSMs (peptide spectrum match) were counted. In the PSM sequence, the phosphorylated residues are shown in red. Note that the phosphorylation at T269 was observed in only one peptide (bold & red)

**Table 2:** The frequency of phosphorylated sites in all phosphorylated AQP2 PSM

Phosphorylated residue	Number of PSM	%
T244	6	3
S256	154	83
S261	14	8
S264	3	2
S269	2	1
Total	185	100

Human urine exosomes were digested by trypsin. Three different samples were examined, and each sample was analyzed thrice by LC–MS/MS. The results of all phosphorylated PSM are summated

The very rare phosphorylation at T269 could be due to either the rare phosphorylation event or to the shorter pT269-containig PMS insufficient for LC–MS analysis. The full trypsin digestion will generate four residue peptides, R_267_GTK_270_, which are too short to be analyzed by LC–MS. To overcome this limitation, we used another protease, Glu-C digesting at residue of E or A to generate peptides of L_259_HSPQSLPRG**T**KA_271_ from human AQP2, which contain T269 with sufficient length of peptides to be identified by LC–MS. Table 3 summarized the results of three exosome samples digested by Glu-C. A total of 47 AQP2 C-terminal phosphorylated PMSs were detected; 46 were peptides between L259 and A271, and one was between V251 and A271. On the other hand, the number of non-phosphorylated PSMs was 78, indicating that 60% of PSMs were phosphorylated. Among the phosphorylated PMSs, the phosphorylation at S261 was much more (81%) than at S264 (19%). Of note, no T269-phosphorylated PMS was detected. Thus, the phosphoproteomic analysis of human urine AQP2 digested by trypsin or Glu-C indicated that S256-phophorylayion was most abundant followed by S261-phophorylayion although T269-phophorylayion was negligible in human AQP2 in urinary exosomes.

**Table 3:**
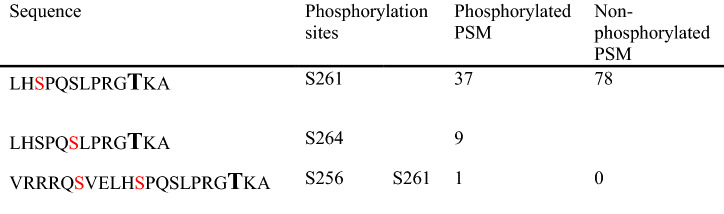
The numbers of phosphorylated and non-phosphorylated T269-containing AQP2 PSM generated by Glu-C digestion from urine exosomes

The phosphorylated residues are shown in red. Note that no phosphorylation at T269 (bold) was observed in three different samples

### Immunoblots with phosphorylated-AQP2 specific antibodies

**Fig. 1:**
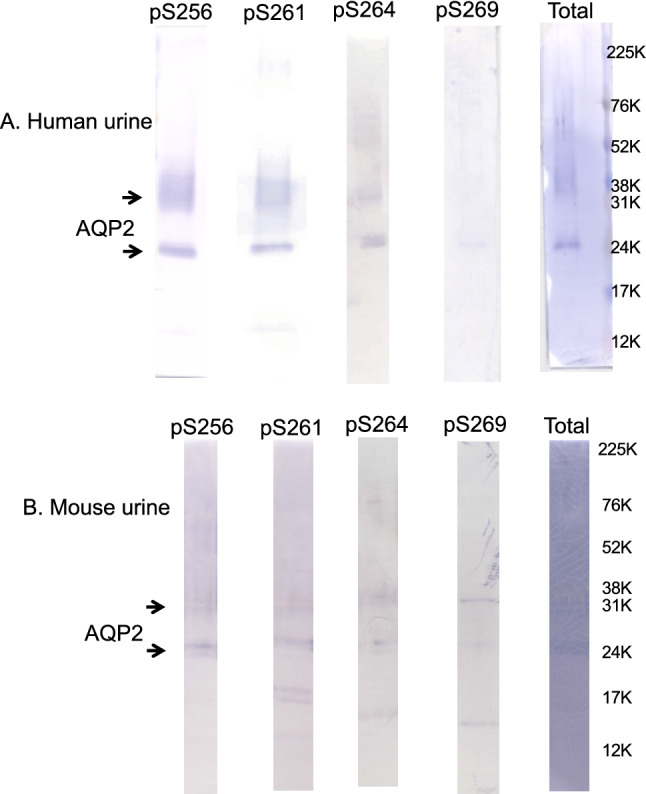
Western blots of urinary exosomes with total-, pS256-, pS261-, pS264- and pS269-AQP2 specific antibodies. The same amounts of urinary exosome samples prepared from human (**a**) and mouse (**b**) were loaded in each lane

To confirm the above phosphoproteomic results, human urinary exosome samples were analyzed by Western blot with phosphorylated-AQP2 specific antibodies. As shown in Fig. 1a, an anti-total-AQP2 antibody detected a band at 25 kDa (non-glycosylated AQP2) and a broad band between 35–45 kDa (glycosylated AQP2) as previously observed for the typical AQP2 immunoblotting. Similar bands were detected by anti pS256-, pS261- and pS264-AQP2 specific antibodies. On the other hand, with the anti pS269-AQP2 specific antibody the bands were minimally visible. Since the antibody against pS269-AQP2 in rodents may not detect pT269-AQP2 in human, we then examined mouse urine exosomes to detect the phosphorylated AQP2 whose residue at 269 is S. As shown in Fig. 1b, a similar staining pattern was observed as was the case in human urinary exosomes, indicating that pS269-AQP2 was also minimally present in mouse urine. These data indicate that 256-, 261-, and 264-phosphorylated AQP2 are excreted in urine, while 269-phosphorylated AQP2 is rarely excreted.

### The effect of VP administration to a central diabetes insipidus patient

**Fig. 2:**
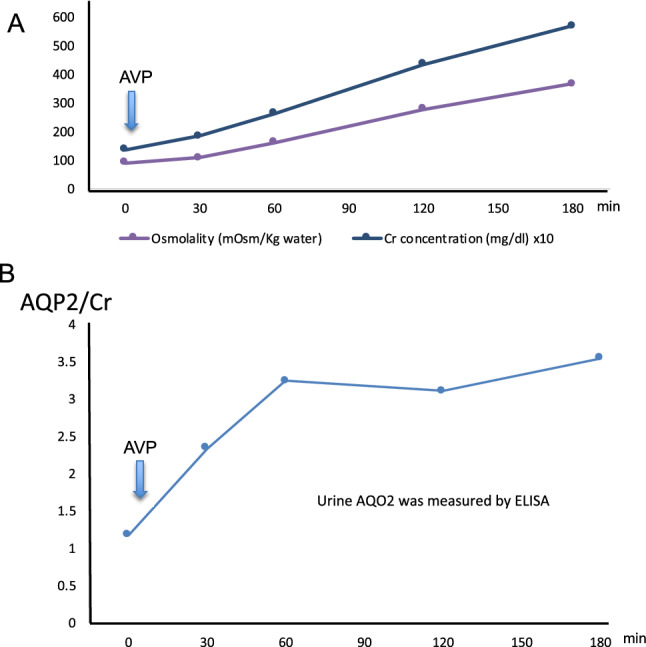
Effects of dDAVP administration in a CDI patient. A. Urine osmolality and creatinine concentrations. B. Urinary AQP2 excretion was corrected by urinary creatinine (AQP2 concentration/creatine concentration)

**Fig. 3:**
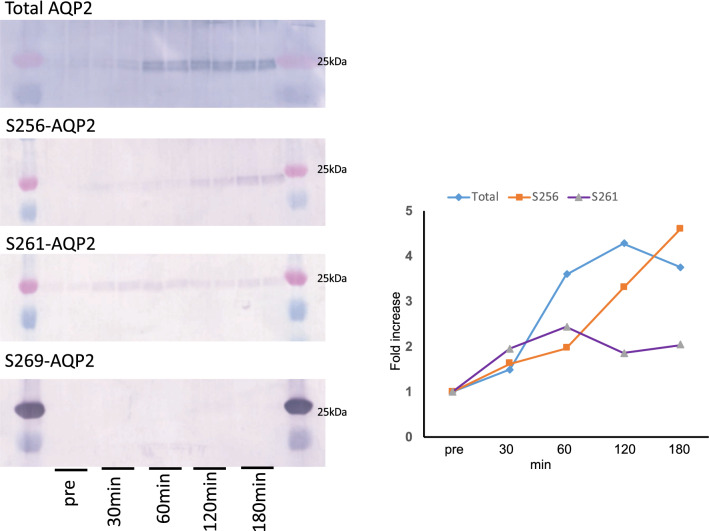
Western blots of urinary exosomes of CDI patient before and after dDAVP treatment. Left, blots were stained with total-, pS256-, pS261- and pS269-AQP2 specific antibodies. Exosome samples equivalent to 2 µg urine creatinine were applied in each lane with duplicate for each time point. Right, the band intensities of the blots were quantified and summarized. For each time point, the mean of two lanes was used

To get insights into the physiological relevance of the AQP2 phosphorylation in urinary excretion, the phosphorylation profile of urine AQP2 was examined in a CDI patient. After dDAVP administration, urine osmolality and creatinine concentration increased steadily for 3 h, from 90 to 368 mOsm/kg H_2_O and from 13.6 to 57.0 mg/dl, respectively (Fig. 2a). Urinary AQP2 excretion (corrected by urine creatinine concentration) also increased but with a different pattern: a rapid increase for the first 1 h and then plateaued for 2 h (Fig. 2b). To document the phosphorylation profile of AQP2 in the timed urine samples, immunoblot analysis were employed. Figure 3 shows immunoblots stained with total-, pS256-, pS261- and pS269-AQP2 specific antibodies. Exosome samples equivalent to 2 µg urine creatinine were applied in each lane with duplicate for each time point. The density of the bands stained with total-AQP2 antibody continued to increase for 1 h, and then plateaued (Fig. 3), which simulated the AQP2 excretion pattern measured by ELISA (Fig. 2b). The band of pS256 was almost invisible before VP but apparent after VP with steady increase up to 3 h. On the other hand, pS261 bands were already present before VP administration and increased 2.4-fold by VP to peak at 60 min followed by a gradual decrease. However, pT269 bands were unidentifiable throughout the study even after a longer exposure (Fig. 3).

## Discussion

Phosphorylation/dephosphorylation at multiple phosphorylation sites in the C-terminal of AQP2 plays a major role in the regulation of AQP2 trafficking [4, 7, 17, 18]. The phosphorylation profile of AQP2 has been extensively examined in rodents, but rarely in humans as human kidney samples are not easily available. In this study, we examined the phosphorylation profile of AQP2 in human urinary exosomes which may reflect that of human kidney tissues.

We successfully showed that all four consensus phosphorylation sites in the C-terminal of human AQP2 (S256, S261, S264, T269) were indeed phosphorylated (Tables 1, 2, 3). However, a previous study by Knepper and coworkers using a similar phosphoproteomic analysis of human urinary exosomes showed the phosphorylation at only a single site, S256 [19]. The enhanced detection sensitivity of our MS machine may explain this discrepancy. It should be stressed that T269 in human AQP2 was phosphorylated in our study. As the phosphorylation at S269 in rodent AQP2 has been shown to be a critical determinant for the apical accumulation of AQP2 [6, 7], the phosphorylation at T269 in human AQP2 may similarly be important as discussed below.

The amount of pS269-AQP2 becomes more abundant at the apical membrane and less in the cytoplasm after VP stimulation in rodents [6]. Accordingly, pS/T269-AQP2 is regarded as the major molecule responsible for VP-mediated water permeability of the collecting duct [4, 9]. Phosphorylation at S/T269 works in two ways: to increase the exocytosis of AQP2-containing intracellular vesicles to the apical membrane, and to decrease the endocytosis of AQP2 from the apical membrane to endosomes [4, 6, 7, 9, 17]. On the contrary, dephosphorylation of pS269 will allow its endocytosis from the apical membrane, leading to the dephosphorylated status at S/T269 of the endocytosed AQP2.

**Fig. 4:**
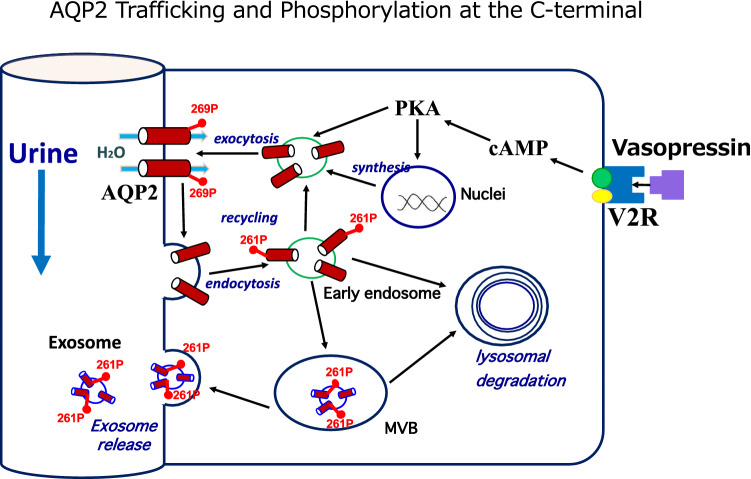
Differential roles of phosphorylations at the C-terminal of AQP2 in its trafficking. Only the phosphorylations at S261 and T269 are marked with red filled circles

Figure 4 illustrates the relationship between AQP2 phosphorylation and its trafficking. The phosphorylations at S261 and T269 are indicated by red filled circles, while those at S256 and S264 are not marked as the constitutively high phosphorylation at S256 is a master switch for phosphorylation of other sites [6, 7, 9] and the very low amount of pS264-AQP2 has unknown significance [9]. The AQP2 located at the apical membrane is phosphorylated at T269 as a signal for insertion and retention at the apical membrane. Subsequently, pT269-AQP2 will be dephosphorylated to be endocytosed into endosomes followed by either recycling or transfer to MVB/lysosomes. Accordingly, AQP2 in the endosomes and MVB is expected to be non-phosphorylated at T269. As exosomes are created from endosomes through the endosome-MVB pathway mediated by ESCRT system [10, 12, 19], AQP2 in exosomes should be the T269-dephosphorylated form (Fig. 4). Therefore, our observation of minimal abundance of pT269-AQP2 in exosomes by phosphoproteomic analyses (Tables 1, 2, 3) and immunoblots (Figs. 1a, b, 3) would suggest the importance of phosphorylation/dephosphorylation at S/T269 as a signal for the apical membrane insertion/internalization of AQP2.

Furthermore, the rare abundance of pS269 in urinary exosomes suggests that urinary AQP2 is not derived directly from the apical membrane (membrane shedding or ectocytosis) as AQP2 in the apical membrane is phosphorylated at this site (Fig. 4). A recent study by Ikeda et al. reported a faint band for pS269-AQP in rat urinary exosomes by Western blot [20]. However, in our similar study (Fig. 1b), we could not detect the band. Further studies will be necessary to resolve this discrepancy although the difference of the antibodies used may be one explanation.

The relatively high abundance of pS261-AQP2 in urinary exosomes (Tables 1, 2, 3) is also noteworthy. Our phosphoproteomic analysis using trypsin-digested samples showed that the frequency order was pS256 >  > pS261 > pS264 > pT269. This order is similar to that in rat inner medulla tissue without dDAVP administration; the percentage of each phosphorylated-AQP2 to the total AQP2 was 22, 18, 1, and 0.1% for pS256, pS261, pS264, and pS269, respectively [9]. Based on these data, the relatively high abundance of pS261-AQP2 in urinary exosomes may reflect the relative abundance in the cells. However, the same study reported a dramatic decrease of pS261 from 18 to 2% and a simultaneous increase of pS269 from 0.1 to 5% in the renal medulla of dDAVP-treated rats [9]. Although this decrease in pS261-AQP2 after VP has also been reported using pS261-AQP2 specific antibodies, its mechanism remains to be clarified [3, 8]. The authors suggested that majority of intracellular pS261-AQP2 is localized in early, late, and recycling endosomes as well as secretory vesicles [8]. In this context, our observation in a CDI patient is consistent with the above observation in that urinary excretion of exosomal pS261-AQP2 transiently increased in 30–60 min after dDAVP administration (Fig. 3). The decrease in cellular pS261-AQP2 may be caused by the stimulated excretion of pS261-AQP2 to the urine. We speculate that the phosphorylation at S261 together with the phosphorylation at 256 may be a signal for AQP2 excretion through exosomes to the urine (Fig. 4).

Why the disposal of AQP2 should be stimulated after VP stimulation? One speculation will be that a stimulated disposal of the aged AQP2 damaged during the recycling is necessary to make a room for the fresh AQP2 to sustain the recycling. However, we previously showed that urinary exosomes have a functional water channel activity mostly from AQP2 [12]. Thus, the urinary exosomes may deliver functional AQP2 to the downstream tubules to enhance the water permeability of the inner medullary collecting duct. Obviously, further studies are needed for understanding the physiological roles of exosomal AQP2 with its relevant phosphorylation profile for its regulated excretion.
